# Evaluating the Nutritional and Immune Potentiating Characteristics of Unfermented and Fermented Turmeric Camel Milk in Cyclophosphamide-Induced Immunosuppression in Rats

**DOI:** 10.3390/antiox11040792

**Published:** 2022-04-18

**Authors:** Thamer Aljutaily

**Affiliations:** Department of Food Science and Human Nutrition, College of Agriculture and Veterinary Medicine, Qassim University, Buraydah 51452, Saudi Arabia; thamer.aljutaily@qu.edu.sa

**Keywords:** turmeric, probiotics, fermented camel milk, polyphenols, immune-boosting, antioxidative stress

## Abstract

Antioxidative, nutritional, and immune-boosting characteristics of turmeric-camel milk (TCM) and fermented turmeric-camel milk (FTCM) were investigated. A cyclophosphamide-induced immunosuppression rat model consisting of six experimental groups was carried out to study the effects of TCM and FTCM on weight gain, antioxidant status, immunoglobulin (Igs), pro-inflammatory and anti-inflammatory cytokines, and oxidative stress biomarkers. TCM or FTCM were orally administrated at 10 or 20 mL Kg^−1^ rat weight to CYP-immunosuppressed rats for 2 weeks in the presence of negative (NR) and positive (CYP) control groups. The phytochemical analysis and antioxidant capacity results indicated that TCM and FTCM contained considerable phenolic content with super antioxidant activities. CYP injection affected the rats’ weight directly during the first week and then, a low weight gain percentage was recorded in treated groups at the end of the experiment. The most efficient treatment for recovering rats’ weight was administering TCM and FTCM at 20 mL kg^−1^. Feed efficiency significantly increased with feeding TCM and FTCM in a dose-dependent manner. A significant improvement was found in WBCs, lymphocytes, and neutrophils count, suggesting that both TCM and FTCM alleviated the CYP-induced immunity suppression in a dose-dependent manner. IgG, IgA, and IgM concentrations in the CYP + TCM at 10 or 20 mL kg^−1^ and CYP + FTCM at 10 or 20 mL kg^−1^ groups were increased significantly. Concentrations of IL-1 beta, IL-6, IL-10, IL-13, and IL-TNF-α in the CYP group were significantly lower than in the NR group. Interestingly, both TCM and FTCM, especially with high doses, significantly enhanced cytokines production. Administrating FTCM was more potent than TCM, indicating that TCM with probiotics fermentation potentiated the immunological activity in immunosuppressed rats. Treated rats with TCM and FTCM can reverse CYP inhibition of antioxidant enzyme activities, significantly increase GSH, CAT, and SOD, and decrease MDA levels in a dose-dependent manner. In conclusion, these observations indicated that FTCM exhibits better improvements in weight gain, increased immune biomarkers in terms of WBCs, enhanced pro-inflammation and anti-inflammation responses, and accelerated antioxidant activity in immunosuppressed rats compared with TCM. It could be beneficial and profitable for boosting immunity and protecting against oxidative stress.

## 1. Introduction

Recently, awareness of consuming functional foods has rapidly increased worldwide. Functional foods strengthen physiological functions, enhance life expectancy, and prevent infections [1]. The worldwide market for functional foods is projected to be valued at about USD 33 billion [2]. The classification of functional foods is (A) improving the steady stomach and colon functions through microbiome (prebiotics and probiotics) or rally children’s life, (B) reducing health risks such as high blood pressure, high cholesterol level, and (C) providing special foods for specific uses such as gluten-free or lactose-free products [3]. Eating foods that provide immunological advantages or over-supplementation is not novel. It is thought to have the potential to reduce inflammation and reduce the impact of illnesses [4,5,6]. Immune-boosting nutrients are a type of nutrient that improves the immune system’s health. Some nutrients, including omega-3 fatty acids, glutamine, arginine, nucleotides, and probiotics, have significantly impacted immune system response [7]. They have also baptized immune nutrients or immunity controllers [8]. Functional food components are potentially beneficial components that originate logically in foods or are added to them as active elements and include vegetables and fruits, whole cereal grains, fortified foods, functional beverages, vitamins, minerals, dietary fiber, fatty acids, polyphenols, phytosterols, stanols, polyols, symbiotic, phytoestrogens, and functional plant-based proteins [7,9].

Furthermore, the health properties of these constituents may be attributable to their offering benefits other than required nourishment. They may reduce or minimize the occurrence of severe illnesses and other health conditions [7,9,10,11]. Among these functional foods is fermented milk, which is probiotic and contains essential amino acids that boost immune function [12,13]. Most spices and seasonings are rich in phytochemicals, antioxidants, minerals, and antimicrobials, which could be beneficial when incorporated into milk or fermented milk to create functional dairy products [14,15].

The association of camel milk (CM) with health benefits may be partly underlined by its functioning as food for the gut microbiota [12,16]. CM has a high overall nutritional value and heat stability [17,18]. It is unknown whether CM can improve microbial community diversity in humans [19]. The effect of CM and fermented camel milk (FCM) consumption on the growth of Clostridiales and the genus Anaerostipes, as well as its association with increased production of short-chain fatty acids (SCFAs) in the gut, on the immune response, has received little attention [12,19,20].

Natural food additives such as turmeric, cinnamon, cardamom, and cloves are remarkably used as functional additives, which might improve nutritional health and immunity [20,21]. Bioactive compounds derived from plants have many potent activities, including antioxidant, anti-diabetic, anti-cancer, antimicrobial, anti-inflammatory, and immunostimulatory impacts [20,22]. Immunostimulant plants or their by-products contain various phenolic, alkaloid, polyphenolic, quinine, terpenoid, lectin, and polypeptide compounds, many of which are powerful alternatives to antibiotics, pesticides, vaccines, and other synthetic compounds [20]. Turmeric (*Curcuma longa* L.) is a plant known for its application for food seasoning practical and medicinal uses [23,24,25]. In herbal and traditional medicine, turmeric treats various diseases. The predominant bioactive compound is curcumin, studied extensively due to its comprehensive medicinal properties but not for immunity boosting [25,26]. Cinnamon bark powder has been scientifically verified and has several biological activities and health benefits as a rich source of natural antioxidants [24,27]. Ginger has been commonly used as a seasoning or traditional condiment for various foods and beverages. Phytochemical studies show that ginger has antioxidant and anti-inflammatory activity, and some of them have the potential for cancer prevention [24,28]. The most recently produced turmeric-fortified soya milk showed the highest protein, iron, zinc, TPC, and antioxidant activity, which present a cheap and nutritious source of golden milk [29].

Recently, obtained finding using a mouse model noticed that consumption of CM results in microbial community alteration better than both cow and goat milk [9,10,12,19,20]. The study of immune-enhancing effects of natural products and their derivatives represents an active area of current research [30,31,32,33,34]. Immunomodulatory functions of numerous plant-based derivatives [11,31,35,36,37] and camel milk [10] have been approved. Furthermore, the research has confirmed the beneficial health impacts of fermented dairy foodstuffs, such as yogurt and cheese, including regulation of food intake, satiety, and obesity-related metabolic disorders [12,13]. However, fermented dairy products such as fermented CM incorporated turmeric and additional food additive have not been studied. Suppose CM or FCM, which combine natural food additives (such as turmeric, cinnamon, cardamom, and clove), increase the abundance of the beneficial microbial community, promote the nutritional status, and enhance the immune response. In that case, it will be important findings that provide critical evidence supporting supplementation with beneficial bacteria and CM/FCM for improving the body’s defense system and enhancing immunity.

Indeed, comprehensive strategies that strengthen the consumption of TCM or FTCM can have nutritious and immune-potentiating health benefits by increasing the gut microbiota diversity accompanied by the increased microbial production of SCFAs in the gut and expanding the defense antioxidant system in the body. Therefore, this proposed proposal is innovative since it takes advantage of plant-based additives and FCM consumption to comprehensively investigate where the nutritious and immune potentiating potential of produced functional CM impact can be significant. Therefore, the current study has been developed to test the central hypothesis of the proposed research with two aims. Firstly, to study the efficacy of TCM or FTCM consumption on improving the nutritional status as improves blood profile, antioxidant status, weight gain. Secondly, to study the impact on immune potentiating properties throughout immunoglobulins and some pro-inflammatory and anti-inflammatory cytokines and oxidative stress biomarkers.

## 2. Materials and Methods

### 2.1. Ingredients

The Fresh CM was obtained from the College of Agriculture and Veterinary Medicine Farm, Qassim University, from October to November 2021. Standard yogurt culture containing a mix of *Streptococcus thermophiles*, *Lactobacillus acidophilus,* and *Bifidobacterium bifidum* strains in freeze-dried direct-to-vat set form (DVS) kept at −18 ± 1 °C was obtained from Christian Hansen (Copenhagen, Denmark). Dry turmeric powder (*Curcuma longa*), ginger rhizomes powder (*Zingiber officinale* L.), cinnamon bark powder (*Cinnamomum burmannii* L.), clove pods (*Syzygium aromaticum*), cardamom (*Elettaria cardamomum*), and pepper (*Piper nigrum*) were obtained from spices local markets (Aba Al-khail, https://jiadalqassim.com/en/, accessed on 1 January 2021) at Buraydah city, Qassim region, Saudi Arabia. All spices have been kept at 4 ± 1 °C in a dry place until further use.

### 2.2. Preparation of Turmeric Camel Milk (TCM) and Fermented Turmeric Camel Milk (FTCM)

The fresh camel milk was chemically analyzed before subjecting it to manufacture. Four letters of camel milk (8.14 g solid not fat, 2.70 g fat, 2.98 g protein, 0.66 g Ash, 4.47 g Lactose 100 mL^−1^ fresh milk) was heated at 50 °C. Subsequently, weighed ingredients (2 g turmeric powder, 5 g honey, 1 g cinnamon powder, 1 g ginger powder, 0.1 g cardamom powder, 0.05 g clove powder, 0.05 g white pepper powder per 100 mL^−1^ fresh milk) were added successively to warm milk, vigorously mixed using a bench-top food processor (Santos, VITA-MAX CORP-Light Industrial Food Preparing Machine Model, VM0122E, Santos Houston, TX, USA) for 2 min at speed 4. The whole mixture was reheated at 50–60 °C and kept for 10 min to extract soluble bioactive compounds in added spices. The prepared TCM was filtered through cheesecloth to remove sediments, divided into two equal portions (2 L each), filled in scraw-capped glass bottles, and pasteurized at 85 °C for 15 min, then cooled down to 42 °C. For FTCM preparation, 1 g per 1 L from ABT-5 starter was added to prepared TCM under aseptic conditions, then incubated at 42 °C for 4–5 h to achieve a pH of 4.6–4.7 before being cooled for 12 to 18 h. The second portion of prepared TCM was kept without fermentation at 4 ± 1 °C. Aseptically, samples (50 mL) were collected from FTCM in sterile bags for microbiological analysis (only to check the viable bacterial count, data not presented).

### 2.3. Determination of Total Phenolic Content (TPC), Total Carotenoids (TC), Total Flavonoids (TF), and Total Flavonols (TFL) in TCM and FTCM

The TPC in TCM and FTCM was determined using Folin–Ciocalteu reagent, according to Yawadio Nsimba et al. [38] was carried out. Briefly, an appropriate freeze-dried sample (CHRIST, Alpha 1–2 LD plus, Osterode am Harz, Germany, 0.032 mbar, −52 °C) was extracted 3 times with 70% methanol. Aliquots of clear supernatant were mixed with (1:10) diluted Folin–Ciocalteu reagent for 5 min and then, Na_2_CO_3_ (7.5%) was added to stop the reaction. After 60 min, the optical density (OD) was measured and compared to the standard curve of gallic acid (GA) solution (*R*^2^ = 0.99). TPC content was expressed as milligrams of gallic acid equivalents (GAE) per 100 g (mg of GAE 100 g^−1^ dw). To determine the total carotenoids (TC), 1 g of the freeze-dried sample was repeatedly extracted with a mixture of acetone and petroleum ether (1:1, *v/v*), according to Yuan et al. [39]. The upper phase will be collected, washed several times with water, and combined with crude extracts. The petroleum ether will be added to the solution to prepare a known volume. TC content has been spectrophotometrically determined at 451 nm as mg 100 g^−1^ dw. The TF and TFL contents were determined according to Mohdaly et al. [40] at 420 and 440 nm with minor modification, respectively. The TF content was determined in the methanolic extract. Aliquots of clear extract were mixed with AlCl_3_ (2%) and kept in the dark for 60 min; then, OD was measured at 420 nm. The TFL was determined by mixing aliquots of methanolic extracts with sodium acetate (5%). After 5 min, AlCl_3_ (2%) was added, kept in the dark for 150 min, and then OD was measured at 440 nm. The content of TF and TFL were expressed as mg quercetin equivalent (QE) per 100 g^−1^ (mg QE 100 g^−1^).

### 2.4. Antioxidant Capacity Determination

Radical scavenging activity was measured spectrophotometrically based on the bleaching of DPPH radicals in purple solution according to Yawadio Nsimba et al. [38]. The DPPH radical scavenging activity percentage was used to plot the Trolox calibration curve. The antiradical activity was expressed as micromoles of Trolox Equivalents (TE) per gram (µmol TE g^−1^). The radical scavenging activity (RSA) of TCM and FTCM against the stable ABTS (2,2′-azino-bis(3-ethylbenzothiazoline-6-sulphonic acid)) radical cation was measured using the method of Al-Qabba et al. [5]. A Trolox calibration curve was plotted as a function of the ABTS radical cation scavenging activity percentage. The final results were expressed as micromoles of Trolox Equivalents (TE) per gram (µmol of TE g^−1^). The chelating activity of TCM and FTCM was measured as protocoled by Zhao et al. [41]. The inhibition % of ferrozine-Fe^2+^ complex creation as metal chelating action was calculated and presented as (mg mL^−1^) when ethylenediaminetetraacetic acid (EDTA) as a positive control was used.

### 2.5. Animals and Experimental Design

Wistar male rats (48 adult rats) weighing between 150–175 g were used in the present investigation. All experiments were approved by the Institutional Animal Ethics Committee (IAEC) of Qassim University, KSA, regulated by the Purpose of the Control and the Supervision of Experiments on Animals (CPCSEA.), Committee under the National Committee of Bioethics (NCBE), Implementing Regulations of the Law of Ethics of Research on Living Creatures. Animals were housed in air-conditioned polypropylene cages under standard laboratory conditions at 25 ± 1 °C for a week for acclimatization. Rats were fed a regular diet and water ad libitum. Subsequently, the rats’ boxes were divided into six groups (*n* = 8/group). Group 1: normal rats did not receive treatment—only 10 mL kg^−1^ body weight intragastric distilled water (NR). For testing immune potentiating, rats were injected (i.p.) with Cyclophosphamide (CYP) at 250 mg Kg^−1^. The treated groups were classified as follows. Group 2: CYP rats fed intragastric distilled water (CYPR). Group 3: CYP rats fed TCM at 10 mL TCM kg^−1^ body weight (TCM10). Group 4: CYP rats fed TCM at 20 mL TCM kg^−1^ body weight (TCM20). Group 5: CYP rats fed FTCM at 10 mL FTCM kg^−1^ body weight (FTCM10). Group 6: CYP rats fed FTCM at 20 mL FTCM kg^−1^ body weight (FTCM20). The TCM and FTCM have been calculated according to Reagan-Shaw et al. [42]. The animal dose of 10 mL^−1^ kg rat is equal to half a serving of TCM or FCM, while the animal dose of 20 mL^−1^ is equal to a serving of TCM or FCM. The rats were weighed individually on the first day of the experiment, marked, distributed to different groups, and weighed at the end of the investigation. The cumulative weight gain was calculated [43]. At the end of the 2nd week, the 12 h-fasted rats were anesthetized, blood samples were collected from the heart puncture of all the animals, and the serum was separated by centrifugation. Clear serum was separated and kept at −18 ± 1 °C for the screening of biochemical analysis and antioxidant markers. Subsequently, animals were humanely sacrificed by cervical dislocation. The abdomen was opened, and the cardiac and pyloric regions were ligated. Spleen, liver, and kidneys were excised and immediately washed with phosphate buffer, dried with paper tissues, and weighed [43].

#### 2.5.1. Measurement of Hematological Parameters

These analyses were conducted as previously described [44] with minor modification. Briefly, around five hundred microliters of blood were collected in a 1.5 mL^−1^ purple capped tube incorporated disodium salt of ethylenediaminetetraacetic acid (EDTA-2Na) from the tail vein of rats at the end of 2nd week. The numbers of white blood cells (WBCs), lymphocytes, and neutrophils were measured using a CBC colter (Mindray BC-2800 vet, Shenzhen Mindary Bio-Medical Electronics Co., LTD, Shenzhen, China).

#### 2.5.2. Immunoglobulin, Pro-Inflammation, and Anti-Inflammation Cytokines Assay

The quantitative analysis of IgG (ab189578, Rat IgG ELISA Kit, Tokyo, Japan), IgA (ab157735, Rat IgA ELISA Kit, Japan), and IgM (ab157738, Rat IgM ELISA Kit, Japan) was determined according to ELISA-based techniques according to kit instructions. Some cytokines for determining pro-inflammation such as IL1-β (ab255730, Rat IL-beta ELISA Kit, Japan), TNF-α (ab236712, Rat TNF-α ELISA Kit, Japan), and anti-inflammation such as IL10 (ab214566, Rat IL10 ELISA Kit, Japan), IL-13 (ab269547, Rat IL-13 ELISA Kit, Japan), and IL-6 (ab234570, Rat IL-6 ELISA Kit, Japan) were examined according to descriptive kit instructions.

#### 2.5.3. Oxidative Stress Biomarkers

Reduced glutathione (GSH, µg dL^−1^) was estimated using GSH colorimetric assay kit (E-BC-K030-S, Elabscience, Houston, TX, USA) according to the method described by Beutler et al. [45]. Lipid peroxidation was estimated using a malondialdehyde (MDA, nmol mL^−1^) colorimetric assay kit (E-BC-K025-S, Elabscience, Houston, TX, USA) by measuring thiobarbituric acid reactive substance (TBARS) and expressed in terms of MDA content according to Ohkawa et al. [46]. MDA, an end product of fatty acid peroxidation, forms a colored complex reacting with Thiobarbituric acid (TBA). The absorbance of the supernatant was measured at 532 nm, and the results were calculated as nmol mL^−1^. Superoxide dismutase (SOD, U L^−1^) activity using SOD typed activity assay kit (E-BC-K022-S, Elabscience, Houston, TX, USA) was determined according to Giannopolitis and Ries [47]. The color reaction was measured at 550 nm, expressed as U L^−1^. Catalase (CAT, U L^−1^) activity was determined using a CAT activity assay kit (E-BC-K031-S, Elabscience, Houston, TX, USA) according to the method of Aebi [48]. All Oxidative stress markers were determined using a blood chemistry analyzer (HumaLyzer 4000, Human Gesellschaft für Biochemica und Diagnostica mbH, Wiesbaden, Germany).

### 2.6. Statistical Analysis

Statistical analysis was performed using SPSS (Ver. 22.0 for Windows, IBM, Houston, TX, USA). Experimental results were expressed as mean ± SE. Statistical significance was tested with one-way ANOVA followed by a post hoc test, and *p*-values < 0.05 were applied according to Steel et al. [49].

## 3. Results

### 3.1. Phytochemicals and Antioxidant Capacities of TCM and FTCM

The quantitative analysis of TCM and FTCM phytochemicals and related antioxidant activities using DPPH, ABTS radical scavenging, and chelating ability (CA) was performed. As can be seen in Table 1, TPC content in TCM and FTCM was 167.25 and 171.21 mg GAE 100 mL^−1^, respectively. The TC content was 11.28 and 11.98 µg 100 mL^−1^. The TF and TFL contents were 15.85 and 15.98 mg QE 100 mL^−1^ and 9.25 and 10.02 mg QE 100 mL^−1^ in TCM and FTCM, respectively. Moreover, DPPH-RSA and ABTS-RSA were used to evaluate the antioxidant capacities of TCM and FTCM. Results indicated 215.68 and 298.27 µmol TE 100 mL^−1^ in TCM and 208.91 and 301.28 µmol TE 100 mL^−1^ in FTCM for DPPH-RSA and ABTS-RSA, respectively. In addition, the antioxidant activity (AOA) of TCM and FTCM is presented in Table 1. Evaluation of the metal-chelating activity revealed 89.37 and 92.09 mg 100 mL^−1^ for TCM and FTCM, respectively, which seems to be proficient in interfering with Fe^2+^—ferrozine complex formation, indicating its capability to chelate oxidation metals.

### 3.2. Effect of TCM and FTCM Administration on Weight Gain %, Organs’ Weight, Food Intake, and Feed Efficiency in CYP-Induced Immunosuppression in Rats

The weight gain percentage, organ relative weight percentage, food intake, and feed efficiency in CYP-induced immunosuppression in rats were monitored; data are tabulated in Table 2. CYP injection affected the rats’ weight directly during the first week, and then a low weight gain percentage was recorded in treated groups at the end of the experiment. The best efficient treatment for recovering rats’ weight was using TCM and FTCM at 20 mL kg^−1^. Low doses of TCM or FTCM were recorded as the lowest weight gain enhancer compared to the NR group. The TCM and FTCM concentration were related to weight recovery in a dose-dependent manner. CYP as a clinical chemotherapeutic drug exhibits immunosuppressive effects, such as body weight loss and decreasing immune organ index in animal models, as shown in Table 2. Due to its cytotoxic nature, it could cause hepatic damage and urotoxicity associated with internal organs. Significant improvements were found in kidneys, liver, and spleen relative organ weight % after giving TCM and FTCM, especially with 20 mL Kg^−1^. The weight of the liver and kidneys varied significantly across all groups. The TCM20 and FTCM20 groups had the highest liver weight, while the CPY-treated rats’ group had the lowest liver weight with a significant difference.

Similarly, kidney and spleen weight were affected due to CYP injection. The considerable difference in their weights was observed only with a high dose of TCM and FTCM. Consequently, the highest food intake was observed in the NR, followed by TCM and FTCM administrated 20 mL Kg^−1^. Feed efficiency significantly increased with feeding TCM and FTCM in a dose-dependent manner compared to the NR group. A significant reduction in food intake and feed efficiency were noted with CYP injection. Interestingly, FTCM presented better efficiency in growth parameters than TCM compared with the NR or CYP groups.

### 3.3. Effect of TCM and FTCM Administration on Hematological Parameters in CYP-Induced Immunosuppression in Rats

The WBCs, lymphocytes, and neutrophils in CYP-induced immunosuppression in rats are illustrated in Table 3. Immediately before slaughtering, about 0.35 mL of fresh blood from the tail vein was taken and subjected to CBC analysis. WBCs count is an indicator of immunity suppression in rats. As exhibited in Table 3, CYP caused a remarkable decrease in WBCs, lymphocytes, and neutrophils in blood compared with the NR group (*p* < 0.05), indicating that CYP caused severe immune suppression. Administration of TCM and FTCM have markedly attenuated the count of WBCs, lymphocytes, and neutrophils. Interestingly, significant improvements were found with giving TCM and FTCM at 20 mL kg^−1^ (*p* > 0.05), suggesting that both TCM and FTCM alleviated the CYP-induced immunity suppression in a dose-dependent manner. The best improvement was remarked with FTCM; even an enhancement better than the NR group was observed.

### 3.4. Effect of TCM and FTCM Administration on Immunoglobulins in CYP-Induced Immunosuppression in Rats

**Figure 1:**
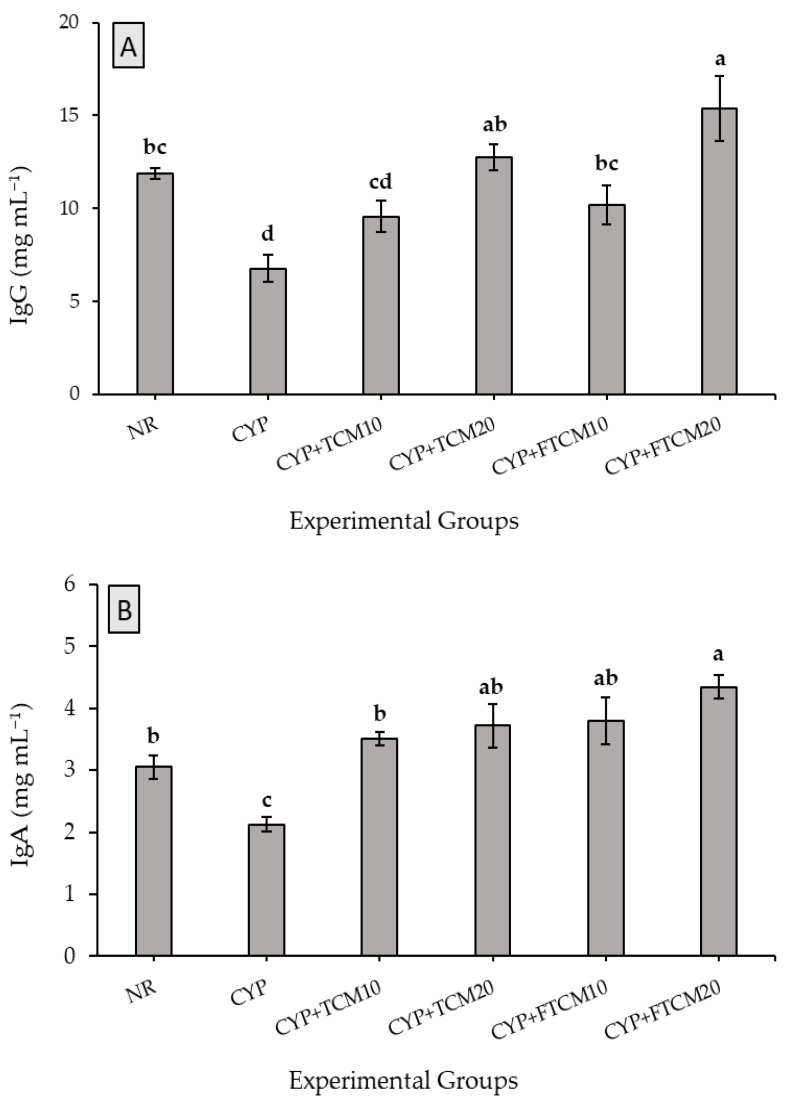

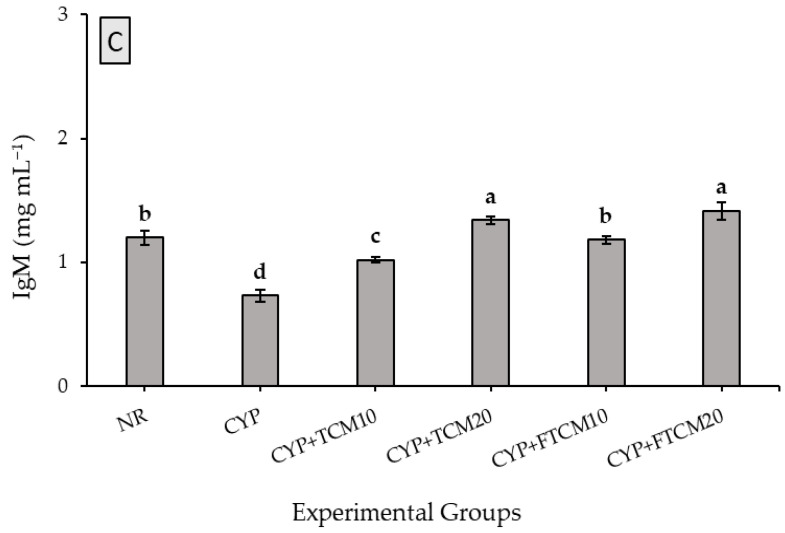
Effects of TCM and FTCM administration on serum IgG (**A**), IgA (**B**), and IgM (**C**) concentrations in CYP-immunosuppressed rats. Results are presented as (mean ± SE, *n* = 8). Bars marked with different letters (a, b, c, and d) indicated statistically significant differences (*p* < 0.05).

Immunoglobulins (Igs) are crucial biomarkers of humoral immunity. IgG, IgM, and IgA are three classes of Igs, which jointly represent almost the total amount of serum Igs [50]. To evaluate the effects of TCM and FTCM administration on humoral immunity in immunosuppressed rats, IgA, IgG, and IgM concentrations in blood serum were measured by related ELISA kits. As illustrated in Figure 1, the IgG, IgM, and IgA concentrations in the CPY group were significantly less than in the NR group, indicating that CYP suppressed humoral immune function (*p* < 0.05). In contrast, the IgG, IgA, and IgM concentrations in the CYP + TCM at 10 or 20 mL kg^−1^ and CYP + FTCM at 10 or 20 mL kg^−1^ groups were increased significantly (p < 0.05). Indeed, giving TCM and FTCM can improve humoral immunity in CYP-immunosuppressed rats in a dose-dependent manner; even production of Igs was accelerated better than the NR group when FTCM was administrated.

### 3.5. Effect of TCM and FTCM Administration on Pro-Inflammation and Anti-Inflammation Cytokines in CYP-Induced Immunosuppression in Rats

Cytokines are essential proteins that can modulate the functions of the T helper cellular [51]. As shown in Table 4, our results showed that the concentrations of IL-1 β, IL-6, IL-10, IL-13, and IL-TNF-α in the CYP group were significantly lower than in the NR group (*p* < 0.05). Compared with the CYP group, the production of determined cytokines was enhanced significantly (*p* < 0.05). Interestingly, both TCM and FTCM, especially with high doses, significantly enhanced Th1 and Th2 cytokines production in CYP-immunosuppressed rats. The enhancement of cytokine production with FTCM was more potent than with TCM, indicating that TCM with probiotics fermentation significantly potentiated the immunological activity in immunosuppressed rats (*p* < 0.05).

### 3.6. Antioxidant Biomarkers

As shown in Table 5, injection of CYP as an immunosuppressive and oxidative stress agent significantly reduced GSH, CAT, and SOD enzymes levels and increased the MDA level in blood serum of CYP-treated rats compared to the NR group. Treated rats with TCM and FTCM at 10 or 20 mL kg^−1^ BW showed remarkable improvement in the activity of antioxidant enzymes GSH, CAT, and SOD, as well as a substantial reduction in MDA levels, were recorded, as shown in Table 5. The improvement rate in GSH was 22.45, 51.46, 45.02, and 74.74% for TCM10, TCM20, FTCM10, and FCTM20, respectively. In the same context, accelerating antioxidant activity rats were 27.04, 64.24, 21.81, 89.95% for CAT and 34.61, 63.00, 62.65, and 88.13% for SOD in TCM10, TCM20, FTCM10, and FCTM20, respectively. The superior attenuation in antioxidants combats the autoxidation process, resulting in MDA reduction by 37.80, 50.23, 39.61, and 52.87% in TCM10, TCM20, FTCM 10, and FCTM20, respectively. These results suggest that administering both TCM and FTCM can reverse CYP inhibition of antioxidant enzyme activities, significantly increase GSH, CAT, and SOD, and decrease MDA levels in a dose-dependent manner. These observations indicate that formulated golden camel milk (GCM) exhibits antioxidant activity in immunosuppressed rats.

## 4. Discussion

The study of immune-enhancing effects of natural products and their derivatives represents an active area of current research [30,31,32,33,34]. Recently, consumers’ increasing preference for natural and healthy foods has contributed to the popularity of turmeric milk, which is now known globally as “golden milk.” Turmeric milk is an example of a traditional drink often consumed to treat a sore throat and as a home therapy for fever [23]. Various ailments such as duodenal ulcer, asthma, malaria, cough, and cold can also be addressed with boiled turmeric milk [20,29]. Commercially, golden milk is formulated from cow’s or plant-based milk, i.e., soybean milk [29]. Since golden milk is widely consumed and its consumption is gaining global popularity. Formulating a camel milk-based drink incorporating mainly turmeric as a fermented and unfermented drink is an excellent idea to attain a natural product with superior protective and therapeutical properties. Claims of combining CM with turmeric and additional ingredients and studying its antioxidant and immunomodulatory properties were targeted in the present work [29].

The valuable phytochemicals content and antioxidant activities in TCM and FTCM indicated rich polyphenols with high antioxidant activity. Turmeric and additional ingredients were added nutritional value and increased biologically active components in TCM. Camel milk is well known to possess antioxidant activity in vitro and in vivo [9,10]. Interestingly, many researchers have confirmed the super antioxidant activity, anti-inflammatory, immune-boosting efficiency of turmeric (*Curcuma longa*) [25,26], ginger (*Zingiber officinale* L.) [24], cinnamon (*Cinnamomum burmannii* L.) [24], clove (*Syzygium aromaticum*), cardamom (*Elettaria cardamomum*), and pepper (*Piper nigrum*) [15]. Biologically active components, such as phenolics, exhibit antioxidant activity by breaking down lipid oxidation chain reactions and supplying hydrogen to active free radicals. The phenolic hydroxyl groups were responsible for phenolics’ ability to scavenge radicals and inhibit them [52,53]. These phenolic acids have been reported as an efficient antioxidant component that inhibits the formation of hydrogen peroxide, hydroxyl radicals, and superoxide anion [54,55]. A direct association exists between increased phenolic component concentration and their antioxidant capacity [56]. The metal chelating activity of TCM and FTCM appears to be capable of interfering with the formation of the “Fe^2+^—ferrozine” complex, implying that it can capture “ferrous” ions before “ferrozine”.

The present results show that CYP as a clinical chemotherapeutic drug exhibits immunosuppressive effects, such as body weight loss and decreasing immune organ index in animal models. Due to its cytotoxic nature could cause hepatic damage and urotoxicity associated with internal organs [32]. Yu et al. [31] indicated a significant body weight loss in the first three days of their experiment. However, oral administration of TCM or FTCM significantly attenuated the body weight, especially in groups administrated 20 mL kg^−1^. This finding confirmed that TCM and FTCM helped to improve the bodyweight recovery of immunosuppressive rates, as mentioned by Zeng et al. [11] and Hussain et al. [57]. Significant improvements were found in kidneys, liver, and spleen relative organ weights after giving TCM and FTCM, especially with 20 mL Kg^−1^ [58,59]. The spleen is a pivotal component of the immune system. The immune organ indexes (important indicators of nonspecific immunity) can reflect the level of immunity moderation to a certain extent [31]. The results indicated a significant attenuation in CPY-treated rats after administration of FTCM or TCM compared with the CYP group. It may be due to the efficiency of GCM and probiotics as immune-boosting agents [31,32,33,34]. Previous studies have also reported dose-dependently increased spleen and thymus indexes [11,31]. Based on the above results, TCM and FTCM could significantly increase the immune organ indexes and body weight, restoring the immune function by repairing the CYP-induced damage to immune organs, as similarly remarked [11,21,26,33,34].

Immunomodulatory functions of numerous plant-based derivatives [11,31,35,36,37] and camel milk [10] have been approved. In the current study, we successfully evaluated the immunomodulatory effects of TCM and FTCM in CYP-induced immunosuppression in a rat model. The high dose of CYP induced the immunosuppression was confirmed by declines in body weight, Igs, and interleukins reduction [11,33,38,39]. IgG antibodies can probably attach to the Fc receptor of macrophages and killer (N) cells to modify the functions of natural killer (NK) cells and macrophages [60]. IgA could promote pathogenic organism adhesion to the mucosal cells and thus display equivalent antiviral and antibacterial activity [61]. IgM could activate the complement system and enhance phagocytosis in the presence of complement and macrophages [62]. T cells are associated with the cell-mediated immune response, whereas B cells are associated with the humoral immune response [63]. Stimulated T cells by antigens were transformed into plasmablasts and then differentiated into plasma cells, secreting immunoglobulin to regulate immune responses [64]. In CYP injected rats, TCM and FTCM induced a significant increase in IgA, IgG, and IgM levels in plasma, which implies TCM and FTCM can enhance the humoral immune response in immunosuppressed rats. These findings were also confirmed with *Curcuma longa* [23,33] and camel milk [9,10].

Interleukins promote the proliferation and differentiation of B lymphocytes and regulate NK cells’ cytotoxic activities [65]. Inflammatory cytokines IL-1β, IL-6, and TNF-α, mainly act on leukocytes and the endothelial cells that form blood vessels to promote and control early inflammatory responses. Activated monocytes and macrophages specifically generate IL-1β, which works with different immune cells, including endothelial cells [66]. TNF-α is an important pro-inflammatory cytokine that produces cytokines such as IL-6. The pleiotropic effects of IL-6, such as improving T cell activation and thymocyte and B-cell differentiation, have been reported [67]. In general, Th1-cytokines enhance the division and proliferation of lymphocytes, facilitate the maturation of DC, and activate Th1-based immune responses [68]. Th2 cytokines, such as IL-6, IL-10, and IL-13, promote Th2-type immune responses and increase antibody production [69]. Our results showed that the IL-1β, IL-6, IL-10, IL-13, and TNF-α concentrations in the CYP group were lower than in the NR group. A significant enhancement in cytokine concentrations was observed in TCM and FTCM, mainly with high doses compared with the CYP group. Thus, TCM and FTCM significantly enhanced Th1 and Th2 cytokines production in CYP-immunosuppressed rats. The enhancement of cytokine production with FTCM was stronger than with TCM, indicating that FTCM potentiated immunological activity in immunosuppressed rats.

Antioxidant enzymes, such as CAT and SOD, and antioxidant peptides, such as GSH, play a critical role in protecting host tissue against oxidative stress initiated by superoxide anions [70]. Lipid peroxidation is a well-recognized oxidative stress mechanism in animal tissues and is generated by reactive oxygen species. MDA is a lipid peroxidation end product involved in forming lipid radicals and oxygen uptake and is a marker for endogenous lipid peroxidation [71]. High doses of CYP cause liver injury and hematologic alterations due to an elevation of oxidative stress [72] associated with excessive ROS production [73]. In the current study, CYP injection markedly decreased GSH, SOD, and CAT and increased MDA levels in the serum of CYP-treated rats compared to NR as well documented [34,36,38]. GSH is a non-enzymatic antioxidant that is found in all mammalian cells. With its oxidized form, GSSG, GSH acts as a cofactor for numerous detoxifying enzymes (GPx, GST, and others) against oxidative stress and maintains cellular redox balance [74]. In the same context, SOD catalyzes the dismutation of two molecules of superoxide anion (* O_2_) to hydrogen peroxide (H_2_O_2_) and molecular oxygen (O_2_), consequently rendering the potentially harmful superoxide anion less hazardous [75]. MDA is the first lipid peroxidation product and is one of the important markers of oxidative stress. ROS increases the risk of tissue damage and causes lipid peroxidation as determined by the catabolite malondialdehyde marker [76]. Administrating TCM and FTCM ameliorated the diverse effects of CYP by restoring the altered activity of antioxidant agents such as SOD, CAT, and GSH and may deactivate the process of producing the MDA [9,10,24,35]. This immunomodulatory efficiency may be due to the potent antioxidative activity of CM and FCM containing turmeric and spices ingredients. Shakeri and Boskabady [33] proved the preventive effect of curcumin on inflammatory cells, inflammatory mediators, oxidative stress, and immunomodulatory effects in rats. As recently confirmed, its more specific immunomodulatory effect caused increased Th1/Th2 balance, with promising therapeutic potential against asthma disease [23]. The possible mechanisms of GCM in their antioxidant activities contain two hypotheses. Firstly, antioxidants could directly mediate free radical activity by interacting with different membrane receptors and/or modulating various post-receptor intracellular signaling pathways [23,33]. Secondly, antioxidants may indirectly exert their antioxidant effects, such as prebiotics, by contributing to the synthesis and release of antioxidants by probiotic bacteria and inhibiting inflammation [33,34].

In our recent study, TCM and FTCM exhibited antioxidant activities in vitro and in vivo. The oral administration of TCM and FTCM showed significant antioxidant activity in vivo, but the detailed mechanism needs further study. Accumulating evidence strongly suggests that antioxidants have immunomodulatory activity [27,28,35]. Moreover, it has been proved that included ingredients could act as prebiotics to promote the growth and metabolism of probiotics. Butyrate, one of the microbial metabolites, could activate immune effector molecules to enhance intestinal mucosal immunity [16]. Meanwhile, intestinal microbiota could modulate oxidative stress by releasing antioxidants [13]. Antioxidants could maintain the membrane fluidity of cells, which is beneficial for them to exert the immune response [77]. Interestingly, camel milk peptides have a strong antioxidant potential that reduces the effects of oxygen free radicals and lipid peroxidation by orchestrating the overall antioxidant system to the optimum in vivo [9]. Moreover, camel milk can reverse CYP-induced leukopenia and weight loss in mice. It may aid in the recovery of crucial antioxidant enzymes that play critical roles in innate immune responses, implying an immunopotentiation effect in diseases or conditions linked with leukopenia or drug-induced toxicity [10].

## 5. Conclusions

Camel milk and medicinal spices have recently gained popularity in functional foods and drinks. A new functional drink containing TCM and FTCM would be created and tested for nutritional and immuno-potentiating properties in CYP-induced immunosuppression in rats. Phytochemical analysis revealed that TCM and FTCM have high phenolic content and exceptional antioxidant activity. TCM and FTCM boosted weight gain %, food intake, and feed efficiency ratio dose-dependently. WBC, lymphocyte, and neutrophil counts were improved significantly, indicating that TCM and FTCM reduced CYP-induced immune suppression. Igs concentrations increased considerably in the CYP + TCM and CYP + FTCM groups at 10 or 20 mL kg^−1^. TCM and FTCM, especially at high doses, increased cytokine production. These treatments can reverse CYP suppression of antioxidant enzyme activity in rats. TCM plus probiotic fermentation potentiated immunological activity in immunosuppressed rats better than unfermented TCM. Interestingly, FTCM improved weight gain, elevated immunological biomarkers such as WBCs and Igs, boosted pro-inflammation and anti-inflammation responses, and increased antioxidant activity in immunosuppressed rats compared with TCM. It could be beneficial and profitable for boosting immunity and defending against oxidative stress damages.

## Figures and Tables

**Table 1:** Total phenolic content, total carotenoids, total flavonoids, total flavonols, and relative potential antioxidant activities of TCM and FTCM (mean ± SE), *n* = 6.

Item	TCM	FTCM
TPC (mg GAE 100 mL^−1^)	167.25 ± 3.15 ^a^	171.21 ± 4.19 ^a^
TC (µg 100 mL^−1^)	11.28 ± 1.15 ^a^	11.98 ± 1.93 ^a^
TF (mg QE 100 mL^−1^)	15.85 ± 1.17 ^a^	15.98 ± 1.38 ^a^
TFL (mg QE 100 mL^−1^)	9.25 ± 0.67 ^a^	10.02 ± 1.12 ^a^
DPPH (µmol of TE 100 mL^−1^)	215.68 ± 6.35 ^a^	208.91 ± 7.09 ^a^
ABTS (µmol of TE 100 mL^−1^)	298.27 ± 9.12 ^a^	301.28 ± 6.28 ^a^
CA (mg 100 mL^−1^)	89.37 ± 3.27 ^a^	92.09 ± 2.24 ^a^

TCM: Turmeric-camel milk, FTCM: Fermented turmeric-camel milk, TPC: Total phenolic content, GAE: Gallic acid equivalent, TC: Total carotenoids, TF: Total flavonoids, QE: Quercetin equivalent, TFL: Total flavonols, TE: Trolox equivalent, ^a^: There is no significant difference (*p* > 0.05) between any two means within the same row have similar superscripted letters.

**Table 2:** Weight gain %, organ’s weight, food intake, feed efficiency of CYP-induced immunosuppression in rats fed different doses of TCM and FTCM (mean ± SE), *n* = 8.

Items	Experimental Groups *					
	NR	CYP	CYP + TCM10	CYP + TCM20	CYP + FTCM10	CYP + FTCM20
Initial BW (g)	195.33 ± 4.48	191.33 ± 3.76	181.83 ± 8.99	187.00 ± 7.80	182.17 ± 4.99	180.17 ± 4.41
Final BW (g)	224.83 ± 3.94	182.67 ± 6.19	192.00 ± 7.45	201.17 ± 7.98	193.67 ± 4.74	195.83 ± 3.46
BW gain %	15.19 ^a^ ± 0.91	−4.62 ± 1.94 ^c^	6.06 ± 2.11 ^b^	7.83 ± 1.26 ^b^	6.43 ± 1.80 ^b^	8.83 ± 1.63 ^b^
Liver weight %	3.12 ± 0.12 ^a^	2.43 ± 0.23 ^c^	2.91 ± 0.21 ^a,b^	3.17 ± 0.26 ^a^	2.89 ± 0.19 ^a,b^	3.29 ± 0.26 ^a^
Kidneys weight %	0.84 ± 0.04 ^a^	0.57 ± 0.00 ^c^	0.59 ± 0.02 ^c^	0.68 ± 0.03 ^b,c^	0.68 ± 0.02 ^b,c^	0.74 ± 0.05 ^a,b^
Spleen weight %	0.68 ± 0.02 ^a^	0.36 ± 0.11 ^b^	0.37 ± 0.06 ^b^	0.57 ± 0.09 ^a^	0.37 ± 0.05 ^b^	0.67 ± 0.09 ^a^
Food intake (g day^−1^) ^#^	17.28 ± 1.54 ^a^	11.01 ± 1.15 ^d^	13.2 ± 1.48 ^c^	16.85 ± 1.58 ^b,c^	14.85 ± 1.67 ^b^	16.91 ± 1.75 ^c^
Feed efficiency ^##^	0.165 ± 0.017 ^a^	0.085 ± 0.006 ^e^	0.125 ± 0.019 ^d^	0.156 ± 0.016 ^b,c^	0.135 ± 0.014 ^a^	0.161 ± 0.011 ^b,c^

SE: Standard error, *: Experimental groups see materials and methods; Section 2.5, BW: Bodyweight, ^#^: Calculated as gram diet/rate per day, ^##^ Feed efficiency = BW gain/food intake, ^a,b,c,d,e^: There is no significant difference (*p* > 0.05) between any two means within the same row have similar superscripted letters.

**Table 3:** Effect of TCM and FTCM on WBCs, Lymphocytes, and Neutrophils in CYP-induced immunosuppression in rats (mean ± SE). *n* = 6.

Hematological Parameters	Experimental Groups *					
	NR	CYP	CYP + TCM10	CYP + TCM20	CYP + FTCM10	CYP + FTCM20
WBCs [10^9^ L^−1^]	8.82 ± 0.07 ^b^	5.90 ± 0.17 ^e^	7.50 ± 0.10 ^d^	9.43 ± 0.20 ^a^	8.15 ± 0.15 ^c^	9.57 ± 0.18 ^a^
Lymphocytes [10^9^ L^−1^]	7.47 ± 0.07 ^b^	4.82 ± 0.20 ^e^	6.50 ± 0.14 ^d^	7.84 ± 0.07 ^a^	7.01 ± 0.09 ^c^	7.94 ± 0.12 ^a^
Neutrophils [10^9^ L^−1^]	1.03 ± 0.03 ^b^	0.95 ± 0.07 ^b,c^	0.80 ± 0.04 ^c^	1.39 ± 0.13 ^a^	0.89 ± 0.04 ^b,c^	1.49 ± 0.04 ^a^

SE: Standard error, *: Experimental groups see materials and methods; Section 2.5, WBCs: White blood cells, ^a,b,c,d,e^: There is no significant difference (*p* > 0.05) between any two means within the same row have similar superscripted letters.

**Table 4:** Effect of TCM and FTCM administration on pro-inflammation and anti-inflammation cytokines in CYP-induced immunosuppression in rats (mean ± SE), *n* = 8.

Cytokines	Experimental Groups *					
	NR	CYP	CYP + TCM10	CYP + TCM20	CYP + FTCM10	CYP + FTCM20
IL-1 β (ng mL^−1^)	58.73 ± 4.62 ^b,c^	33.60 ± 4.78 ^d^	49.78 ± 4.32 ^c^	69.70 ± 6.53 ^b^	66.35 ± 3.44 ^b^	93.63 ± 2.27 ^a^
IL-6 (ng mL^−1^)	196.00 ± 3.58 ^c^	126.67 ± 6.67 ^d^	212.67 ± 9.09 ^b,c^	244.00 ± 4.38 ^a,b^	223.33 ± 5.70 ^b,c^	268.00 ± 22.41 ^a^
IL-10 (ng mL^−1^)	33.72 ± 3.72 ^a,b^	19.94 ± 2.60 ^c^	27.41 ± 2.69 ^b,c^	38.89 ± 2.32 ^a^	33.89 ± 3.55 ^a,b^	42.64 ± 4.14 ^a^
IL-13 (ng mL^−1^)	79.27 ± 1.82 ^c^	52.48 ± 0.47 ^e^	66.07 ± 1.48 ^d^	91.98 ± 1.35 ^b^	78.17 ± 1.04 ^c^	95.93 ± 1.61 ^a^
IL-TNF-α (ng mL^−1^)	95.93 ± 1.61 ^a^	12.41 ± 1.03 ^d^	15.91 ± 1.97 ^c,d^	15.91 ± 1.97 ^c,d^	21.12 ± 2.56 ^b,c^	29.65 ± 2.83 ^a^

*: Experimental groups see materials and methods; Section 2.5, ^a,b,c,d,e^: No significant difference (*p* > 0.05) between any two means within the same row have similar superscripted letters.

**Table 5:** Effects of oral administration of TCM and FTCM on antioxidant biomarkers in CYP-induced immunosuppression in rats (mean ± SE), *n* = 8.

Experimental Groups *	Antioxidant Biomarkers			
	GSH (µg dL^−1^)	CAT (U L^−1^)	SOD (U L^−1^)	MDA (µ mol mL^−1^)
NR	75.02 ± 7.43 ^a,b^	85.44 ± 11.96 ^b^	117.08 ± 3.66 ^b^	25.50 ± 2.25 ^b^
CYP	49.40 ± 4.14 ^d^	59.29 ± 6.20 ^d^	72.98 ± 0.94 ^d^	47.36 ± 4.04 ^a^
CYP + TCM10	60.49 ± 2.32 ^c^	75.32 ± 9.73 ^b,c^	98.24 ± 1.27 ^c^	29.46 ± 3.26 ^b^
CYP + TCM20	74.82 ± 10.18 ^a,b^	97.38 ± 9.13 ^b^	118.96 ± 1.77 ^b^	23.57 ± 3.09 ^b,c^
CYP + FTCM10	71.64 ± 6.98 ^a,b^	72.22 ± 9.12 ^b,c^	118.70 ± 1.46 ^b^	28.60 ± 2.85 ^b^
CYP + FTCM20	86.32 ± 8.98 ^a^	112.62 ± 11.56 ^a^	137.30 ± 1.67 ^a^	22.32 ± 3.56 ^b,c^

*: Experimental groups see materials and methods; Section 2.5, GSH: Reduced glutathione, CAT: Catalase, SOD: Superoxide dismutase, MDA: Malonaldehyde, ^a,b,c,d^: No significant difference (*p* > 0.05) between any two means within the same column with similar superscripted letters.

## Data Availability

Data is contained within the article.

## Abbreviations

ABTS: 2:2′-azino-bis(3-ethylbenzothiazoline-6-sulfonic acid); AOA: Antioxidant activity; CAT, Catalase; DPPH: 1,1-diphenyl-2-picryl hydrazine; dw: Dry weight; FCM, Fermented camel milk; FTCM: Fermented turmeric-camel milk; GA: Gallic acid; GAE: Gallic acid equivalent; GSH: Reduced-glutathione; MDA: Malonaldehyde; QE: Quercetin equivalent; RSA: Radical scavenging activity; SE: Standard error; SOD: Superoxide dismutase; TBA, Thiobarbituric acid; TC: Total carotenoids; TCM: Turmeric-camel milk; TE: Trolox equivalents; TF: Total flavonoids; TFL, Total flavonols; TPC, Total phenolic compounds.

## References

[1] Roberfroid M.B. (2000). Concepts and Strategy of Functional Food Science: The European Perspective. Am. J. Clin. Nutr..

[2] Hilliam M. (2000). Functional Food––How Big is The Market. World Food Ing..

[3] Bigliardi B., Galati F. (2013). Innovation trends in the food industry: The case of functional foods. Trends Food Sci. Technol..

[4] Vanneste J., Cornish D., Yu J., Voyle M. (2002). P10c: A New Biological Control Agent for Control of Fire Blight Which Can be Sprayed or Distributed Using Honey Bees. Acta Hortic..

[5] Al-Qabba M.M., El-Mowafy M.A., Althwab S.A., Alfheeaid H.A., Aljutaily T., Barakat H. (2020). Phenolic Profile, Antioxidant Activity, and Ameliorating Efficacy of *Chenopodium quinoa* Sprouts against CCl4-Induced Oxidative Stress in Rats. Nutrients.

[6] Aleid I.S., Alfheeaid H.A., Aljutaily T., Alhomaid R.M., Alharbi H.F., Althwab S.A., Abdel-Rahman H.A., Algeffari M.A., Barakat H. (2021). Gastroprotective effects of spirulina platensis, golden kiwifruit flesh, and golden kiwifruit peel extracts individually or in combination against indomethacin-induced gastric ulcer in rats. Nutrients.

[7] Guiné R., Lima M., Barroca M. (2009). Role and health benefits of different functional food components. Millenium.

[8] Schloerb P.R. (2001). Immune-Enhancing Diets: Products, Components, and their Rationales/Discussion. JPEN J. Parenter Enteral. Nutr..

[9] Ebaid H., Abdel-Salam B., Hassan I., Al-Tamimi J., Metwalli A., Alhazza I. (2015). Camel milk peptide improves wound healing in diabetic rats by orchestrating the redox status and immune response. Lipids Health Dis..

[10] Khan M.A. (2017). Immune potentiating and antitoxic effects of camel milk against cyclophosphamide-induced toxicity in BALB/C mice. Int. J. Health Sci..

[11] Zeng Y., Hu X., Yu Z., Wang F., Zhang Z., He K., Tian H., Yu F. (2021). Immune Enhancement and Antioxidant Effects of Low Molecular-Weight Peptides Derived from *Nibea Japonica* Muscles on Immune-Deficient Mice Induced by Cyclophosphamide. Process Biochem..

[12] Aljutaily T., Huarte E., Martinez-Monteagudo S., Gonzalez-Hernandez J.L., Rovai M., Sergeev I.N. (2020). Probiotic-enriched milk and dairy products increase the gut microbiota diversity: A comparative study. Nut. Res..

[13] Bengmark S., Gil A. (2006). Bioecological and Nutritional Control of Disease: Prebiotics, Probiotics and Synbiotics. Nutr. Hosp..

[14] London C. (2010). Functional foods that boost the immune system. Funct. Food Prod. Develop..

[15] Shobana S., Akhilender Naidu K. (2000). Antioxidant Activity of Selected Indian Spices. Prostaglandins Leukot. Essent. Fat. Acids.

[16] van Vliet M.J., Harmsen H.J., de Bont E.S., Tissing W.J. (2010). The role of intestinal microbiota in the development and severity of chemotherapy-induced mucositis. PLoS Pathog..

[17] Berhe T., Seifu E., Ipsen R., Kurtu M.Y., Hansen E.B. (2017). Processing Challenges and Opportunities of Camel Dairy Products. Int. J. Food Sci..

[18] Elagamy E.I. (2000). Effect of Heat Treatment on Camel Milk Proteins with Respect to Antimicrobial Factors: A Comparison with Cows’ and Buffalo Milk Proteins. Food Chem..

[19] Solanki D., Hati S. (2018). Fermented camel milk: A Review on its Bio-Functional Properties. Emir. J. Food Agric..

[20] Giri S.S., Sukumaran V., Park S.C. (2019). Effects of Bioactive Substance From Turmeric on Growth, Skin Mucosal Immunity and Antioxidant Factors in Common Carp, *Cyprinus carpio*. Fish Shellfish Immunol..

[21] Abo Ghanima M.M., Elsadek M.F., Taha A.E., Abd El-Hack M.E., Alagawany M., Ahmed B.M., Elshafie M.M., El-Sabrout K. (2020). Effect of Housing System and Rosemary and Cinnamon Essential Oils on Layers Performance, Egg Quality, Haematological Traits, Blood Chemistry, Immunity, and Antioxidant. Animals.

[22] Lopez-Varela S., Gonzalez-Gross M., Marcos A. (2002). Functional Foods and The Immune System: A Review. Eur. J. Clin. Nutr..

[23] Boskabady M.H., Amin F., Shakeri F. (2021). The Effect of *Curcuma longa* on Inflammatory Mediators and Immunological, Oxidant, and Antioxidant Biomarkers in Asthmatic Rats. Evid. Based Complement. Alternat. Med..

[24] Thakur R., Yadav K., Khadka K.B. (2013). Study of antioxidant, ANTIBACTERIAL and Anti-inflammatory Activity of Cinnamon (*Cinamomum tamala*), GINGER (*Zingiber officinale*) and Turmeric (*Curcuma longa*). Am. J. Life Sci..

[25] Jurenka J.S. (2009). Anti-inflammatory properties of curcumin, a major constituent of Curcuma longa: A review of preclinical and clinical research. Altern. Med. Rev..

[26] Salehi B., Stojanović-Radić Z., Matejić J., Sharifi-Rad M., Anil Kumar N.V., Martins N., Sharifi-Rad J. (2019). The therapeutic potential of curcumin: A review of clinical trials. Eur. J. Med. Chem..

[27] Abeysekera W., Arachchige S.P.G., Abeysekera W., Ratnasooriya W.D., Medawatta H. (2019). Antioxidant and glycemic regulatory properties potential of different maturity stages of leaf of ceylon cinnamon (*Cinnamomum zeylanicum* Blume) *in vitro*. Evid. Based Complement. Alternat. Med..

[28] Liu C., Cai D., Zhang L., Tang W., Yan R., Guo H., Chen X. (2016). Identification of hydrolyzable tannins (punicalagin, punicalin and geraniin) as novel inhibitors of hepatitis B virus covalently closed circular DNA. Antivir. Res..

[29] Idowu-Adebayo F., Fogliano V., Linnemann A. (2022). Turmeric-Fortified Cow and Soya Milk: Golden Milk as a Street Food to Support Consumer Health. Foods.

[30] Wang K., Conlon M., Ren W., Chen B.B., Bączek T. (2018). Natural Products as Targeted Modulators of the Immune System. J. Immunol. Res..

[31] Yu F., He K., Dong X., Zhang Z., Wang F., Tang Y., Chen Y., Ding G. (2020). Immunomodulatory Activity of Low Molecular-Weight Peptides from *Nibea japonica* Skin in Cyclophosphamide-Induced Immunosuppressed Mice. J. Funct. Foods.

[32] Feng H., Fan J., Lin L., Liu Y., Chai D., Yang J. (2019). Immunomodulatory Effects of Phosphorylated Radix *Cyathulae officinalis* Polysaccharides in Immunosuppressed Mice. Molecules.

[33] Shakeri F., Boskabady M.H. (2017). Anti-inflammatory, Antioxidant, and Immunomodulatory Effects Of Curcumin in Ovalbumin-Sensitized Rat. Biofactors.

[34] Huang F., Zhang R., Liu Y., Xiao J., Liu L., Wei Z., Yi Y., Zhang M., Liu D. (2016). Dietary litchi pulp polysaccharides could enhance immunomodulatory and antioxidant effects in mice. Int. J. Biol. Macromol..

[35] Shirani K., Hassani F.V., Razavi-Azarkhiavi K., Heidari S., Zanjani B.R., Karimi G. (2015). Phytotrapy of cyclophosphamide-induced immunosuppression. Environ. Toxicol. Pharmacol..

[36] Zheng Y., Li S., Li C., Shao Y., Chen A. (2022). Polysaccharides from Spores of *Cordyceps cicadae* Protect against Cyclophosphamide-Induced Immunosuppression and Oxidative Stress in Mice. Foods.

[37] Noh E.M., Kim J.M., Lee H.Y., Song H.K., Joung S.O., Yang H.J., Kim M.J., Kim K.S., Lee Y.R. (2019). Immuno-enhancement effects of *Platycodon grandiflorum* extracts in splenocytes and a cyclophosphamide-induced immunosuppressed rat model. BMC Complement. Altern. Med..

[38] Yawadio Nsimba R., Kikuzaki H., Konishi Y. (2008). Antioxidant activity of various extracts and fractions of *Chenopodium quinoa* and *Amaranthus* spp. seeds. Food Chem..

[39] Yuan G.F., Sun J., Yuan Q., Wang Q.M. (2009). Effects of different cooking methods on health-promoting compounds of broccoli. J. Zhejiang Univ. Sci. B.

[40] Mohdaly A.A.A., Hassanien M.F.R., Mahmoud A., Sarhan M.A., Smetanska I. (2012). Phenolics Extracted from Potato, Sugar Beet, and Sesame Processing By-Products. Int. J. Food Prop..

[41] Zhao H., Dong J., Lu J., Chen J., Li Y., Shan L., Lin Y., Fan W., Gu G. (2006). Effects of Extraction Solvent Mixtures on Antioxidant Activity Evaluation and Their Extraction Capacity and Selectivity for Free Phenolic Compounds in Barley (*Hordeum vulgare* L.). J. Agri. Food Chem..

[42] Reagan-Shaw S., Nihal M., Ahmad N. (2008). Dose Translation from Animal to Human Studies Revisited. FASEB J..

[43] Kang J.-H., Yun S.-I., Park H.-O. (2010). Effects of Lactobacillus gasseri BNR17 on body weight and adipose tissue mass in diet-induced overweight rats. J. Microbiol..

[44] Lee H.Y., Park Y.M., Lee Y.H., Kang Y.G., Lee H.M., Park D.S., Yang H.J., Kim M.J., Lee Y.-R. (2018). Immunostimulatory Effect of *Zanthoxylum schinifolium*-Based Complex Oil Prepared by Supercritical Fluid Extraction in Splenocytes and Cyclophosphamide-Induced Immunosuppressed Rats. Evid. Based Complement. Alternat. Med..

[45] Beutler E. (1963). Improved Method for the Determination of Blood Glutathione. J. Lab. Clin. Med..

[46] Ohkawa H., Ohishi N., Yagi K. (1979). Assay for Lipid Peroxides in Animal Tissues by Thiobarbituric Acid Reaction. Anal. Biochem..

[47] Giannopolitis C.N., Ries S.K. (1977). Superoxide Dismutases: I. Occurrence in Higher Plants. Plant Physiol..

[48] Aebi H. (1984). [13] Catalase *in vitro*. Meth. Enzymol..

[49] Steel R.G. (1997). Pinciples and Procedures of Statistics a Biometrical Approach.

[50] Hsu E. (2018). Immune System Receptors in Vertebrates: Immunoglobulins. Reference Module in Life Sciences.

[51] Millán O., Brunet M. (2016). Cytokine-based immune monitoring. Clin. Biochem..

[52] Tosun M., Ercisli S., Sengul M., Ozer H., Polat T., Ozturk E. (2009). Antioxidant properties and total phenolic content of eight Salvia species from Turkey. Biol. Res.

[53] Ollanketo M., Peltoketo A., Hartonen K., Hiltunen R., Riekkola M.-L. (2002). Extraction of Sage (*Salvia officinalis* L.) by Pressurized Hot Water and Conventional Methods: Antioxidant Activity of the Extracts. Eur. Food Res. Technol..

[54] Farhat M.B., Chaouch-Hamada R., Sotomayor J.A., Landoulsi A., Jordán M.J. (2014). Antioxidant potential of *Salvia officinalis* L. residues as affected by the harvesting time. Ind. Crops Prod..

[55] Roby M.H.H., Sarhan M.A., Selim K.A.-H., Khalel K.I. (2013). Evaluation of antioxidant activity, total phenols and phenolic compounds in thyme (*Thymus vulgaris* L.), sage (*Salvia officinalis* L.), and marjoram (*Origanum majorana* L.) extracts. Ind. Crops Prod..

[56] Ebrahimzadeh M.A., Nabavi S.M., Nabavi S.F., Bahramian F., Bekhradnia A.R. (2010). Antioxidant and free radical scavenging activity of *H. officinalis* L. var. angustifolius, *V. odorata*, *B. hyrcana* and *C. speciosum*. Pak. J. Pharm. Sci..

[57] Hussain A., Shadma W., Maksood A., Ansari S.H. (2013). Protective effects of Picrorhiza kurroa on cyclophosphamide-induced immunosuppression in mice. Pharmacogn. Res..

[58] Yu F., Zhang Z., Ye S., Hong X., Jin H., Huang F., Yang Z., Tang Y., Chen Y., Ding G. (2019). Immunoenhancement effects of pentadecapeptide derived from *Cyclina sinensis* on immune-deficient mice induced by Cyclophosphamide. J. Funct. Foods.

[59] Zhu G., Luo J., Du H., Jiang Y., Tu Y., Yao Y., Xu M. (2018). Ovotransferrin enhances intestinal immune response in cyclophosphamide-induced immunosuppressed mice. Int. J. Biol. Macromol..

[60] Meulenbroek A. (2008). Human IgG Subclasses: Useful Diagnostic Markers for Immunocompetence.

[61] Mantis N.J., Rol N., Corthésy B. (2011). Secretory IgA’s complex roles in immunity and mucosal homeostasis in the gut. Mucosal Immunol..

[62] Heyman B., Shulman M.J., Ratcliffe M.J.H. (2016). Structure, Function, and Production of Immunoglobulin M (IgM). Encyclopedia of Immunobiology.

[63] Mottet M., Goffinet L., Beckers A., Bodart G., Morrhaye G., Kermani H., Renard C., Martens H., Geenen V. (2011). The Role of the Thymus in the Integrated Evolution of the Recombinase-Dependent Adaptive Immune Response and the Neuroendocrine System. Neuroimmunomodulation.

[64] Valdez Y., Brown E.M., Finlay B.B. (2014). Influence of the microbiota on vaccine effectiveness. Trends Immunol..

[65] Brocker C., Thompson D., Matsumoto A., Nebert D.W., Vasiliou V. (2010). Evolutionary divergence and functions of the human interleukin (IL) gene family. Hum. Genom..

[66] van der Poll T., Opal S.M. (2008). Host–pathogen interactions in sepsis. Lancet Infect. Dis..

[67] Huang M., Yang D., Xiang M., Wang J. (2015). Role of interleukin-6 in regulation of immune responses to remodeling after myocardial infarction. Heart Fail. Rev..

[68] Dillinger B., Ahmadi-Erber S., Lau M., Hoelzl M.A., Erhart F., Juergens B., Fuchs D., Heitger A., Ladisch S., Dohnal A.M. (2018). IFN-γ and tumor gangliosides: Implications for the tumor microenvironment. Cell. Immunol..

[69] Stewart D., Nichol A. (2021). Inflammation, Immunity and Allergy. Anaesth. Intensive Care.

[70] Valko M., Leibfritz D., Moncol J., Cronin M.T.D., Mazur M., Telser J. (2007). Free Radicals and Antioxidants in Normal Physiological Functions and Human Disease. Int. J. Biochem. Cell Biol..

[71] Huang X., Nie S., Cai H., Zhang G., Cui S.W., Xie M., Phillips G.O. (2015). Study on *Dendrobium officinale* O-acetyl-glucomannan (Dendronan^®^): Part VI. Protective Effects Against Oxidative Stress in Immunosuppressed Mice. Food Res. Int..

[72] Alqahtani S., Mahmoud A.M. (2016). Gamma-Glutamylcysteine Ethyl Ester Protects against Cyclophosphamide-Induced Liver Injury and Hematologic Alterations via Upregulation of PPARγ and Attenuation of Oxidative Stress, Inflammation, and Apoptosis. Oxidative Med. Cell. Longev..

[73] Cheng X., Gao D.-X., Song J.-J., Ren F.-Z., Mao X.-Y. (2015). Casein glycomacropeptide hydrolysate exerts cytoprotection against H2O2-induced oxidative stress in RAW 264.7 macrophages via ROS-dependent heme oxygenase-1 expression. RSC Adv..

[74] Valko M., Rhodes C.J., Moncol J., Izakovic M., Mazur M. (2006). Free Radicals, Metals and Antioxidants in Oxidative Stress-Induced Cancer. Chem.-Biol. Interact..

[75] Dringen R., Pawlowski P.G., Hirrlinger J. (2005). Peroxide Detoxification by Brain Cells. J. Neurosci. Res..

[76] Ng S.-C., Anderson A., Coker J., Ondrus M. (2007). Characterization of Lipid Oxidation Products in Quinoa (*Chenopodium quinoa*). Food Chem..

[77] Puertollano M.A., Puertollano E., Alvarez de Cienfuegos G., de Pablo M.A. (2011). Dietary antioxidants: Immunity and host defense. Curr. Top. Med. Chem..

